# Medical and Physical Intelligence

**Published:** 1826-01

**Authors:** 


### MEDICAL AND PHYSICAL INTELLIGENCE.

### MORBID ANATOMY.

1. *Unusual Conformation of the Nervous System in a Cretin.*—In the Medicinische Jahrbucher for 1819, Dr. SCHIFFNER, physician to the Great Civil Hospital of Vienna, related the appearances observed on dissection of a cretin, and which chiefly concerned the nervous system. Since that time, Dr. S. has had occasion to examine the body of another cretin, a brother of the former, and has detailed the particulars in the same work for the year 1821; the appearances very nearly coinciding, we shall content ourselves with giving those of the latter individual, who was thirty-three years of age at the time of his death, the cause of which is not mentioned. The skull cap was unusually thick and heavy. The vessels of the scalp, and the sinuses of the brain, were full of blood. Between the membranes, and in the lateral ventricles of this organ, were several ounces of serum, the cortical substance natural, the medullary somewhat vascular, the convolution of the brain cemented by pretty deep sulci; the thalami optici, the corpora striata, &c. presented nothing unnatural, excepting being somewhat flattened from the compression to which they had been subjected. The four first pairs of nerves exhibited nothing unusual, but the third part of the fifth pair exhibited, in almost all its branches, soon after its separation into branches, ganglia or tubera, the size of garden peas. The only branch of the sixth pair similarly circumstanced, was that which accompanies inferior twig of the vidian nerve into the canalis caroticus; and which formed on either side a ganglion the size of a hazel nut. The portio mollis of the seventh pair was little affected, whereas almost all the branches of the portio dura presented ganglia of the size of peas, and larger. In the eighth pair were only seen some oblong swellings. In the neck, both laryngeal nerves, the branches joining the sympathetic nerve, and several muscular branches, were considerably swollen; in the first, those of the œsophageal, and of the posterior pulmonary plexus, had tubera or ganglia of the size already mentioned, viz. of peas. In the axillary plexus, and particularly in those nervous bundles which give origin to the spiro-muscular nerve, they were of the size of hazel nuts. The other nerves of the plexus, particularly the external and internal cutaneous nerve, were provided with similar swellings; fewer in number, but larger, were the ganglia on the dorsal and lumbar nerves, and the largest of all in the neighbourhood of the crista ilii. The nerves of the inferior extremities exhibited, during their course, several considerable swellings of this kind. The great sympathetic, and its branches, presented nothing unnatural; but its ganglia, in the neighbourhood of the spinal column, had, in several places, attained an unusual size; thus the left sympathetic nerve, opposite to the sixth cervical vertebra, exhibited a ganglion of the size and shape of a hen's egg compressed. These ganglia were an actual degeneration of the medullary substance; they could not be detached from the nerves, nor could the nervous fibres be traced either around or through them. They were of a pale rose red colour, and were distinguished from the rest of the nervous medullary matter by the medullary globules not being so regularly arranged or strung together, but confusedly mixed. All were provided with nutrient vessels, and were enclosed in a covering of neurilem. These swellings, neither in the present individual, nor in his brother, shewed any tendency to induration or ossification, and occasioned no pain, circumstances distinguishing them from all similar nervous malformations. Perhaps they may throw some light on the nature of cretinism. The fact of two brothers, having the same malformation of the nervous system, with a similar state of intellect, goes far in the opinion of Dr. Schiffner to prove that, instead of looking for the cause of cretinism in the external influences to which the individual is exposed, in the water, in the air, its want of electricity, &c. we are rather to consider the degraded condition as dependent on malformation, originating with the very act of generation,—from the very earliest period of vital life.

2. *Unusually small Heart.*—M. Masseau relates the case of a little girl subject to convulsions from her infancy, who died at twelve years of age of epilepsy. On opening the head, nothing remarkable was found, excepting a congestion of blood in the sinuses of the dura mater, and in the vessels of the brain, which was rather softer than usual; but, in the chest, the heart was found to be not larger than a common sized hen's egg, and which was almost entirely made up of the right auricle. The author believes that the right ventricle, not having been able to contain all the blood sent to it from the right auricle, this fluid returned into the vena cava, the jugular veins, and the brain, and thus gave rise to the convulsive attacks. (*Bulletin des Sciences Medicales, Octobre.*)

3. *Tumour of the right Ovarium.*—M. Vettu relates the case of a woman, twenty-five years of age, where this disease came on in consequence of a blow on the abdomen ; it increased for seventeen years, and, finally, killed the patient at the age of forty-two, having acquired the weight of fifty-six pounds. Its substance was homogenous, greyish, of a cartilaginous consistence, excepting at three points where it was softened, and of a substance similar to the brain; for a long time it had only proved inconvenient by its weight, and it only began to affect the health about three months before the patient died. (*Idem.*)

---

### THERAPEUTICS.

4. *Cupping Glasses to Poisoned Wounds.*—Dr. Barry, an English physician, read a paper to the Royal Academy of Medicine, relative to some experiments made by him, tending to prove that cupping glasses applied to a poisoned wound prevents the absorption of the venomous matter. The experiments were as follow:—Wounds were made upon the back and thighs of full-grown rabbits, and, when the blood had ceased to flow, two or three grains of strychnia in powder, or two or three drops of hydrocyanic acid, were introduced into the wounds; then, after intervals of three, five, and ten minutes, a cupping glass was applied to the wound, which was renewed as often as it fell off; no symptoms of poisoning occurred in these animals: but if, on the contrary, this precaution was not taken, they all died. On one occasion, Dr. Barry waited until the animal became affected with convulsions, nevertheless, he succeeded in saving it by these experiments. Dr. Barry, who believes that the circulation of blood in the veins takes place in consequence of an action exercised upon that fluid by the thorax during inspiration, concludes that any circumstance, capable of changing the force of this action from the circumference to the centre in an inverse ratio,—that is, from the centre to the circumference, as is done by the cupping glass, will not only prevent absorption, but will also bring back to the surface the matter already absorbed,—as long, at least, as it remains within the influence of this action. (*Archives Generales,* Sept.)

At a subsequent meeting, M. ADELON read a report of M. LAENNEC upon the experiments of Dr. BARRY, the results of which have been verified:—a cupping glass having been applied upon a wound into which some stychnia in powder had been placed, prevented the effects of this substance from manifesting themselves, and also suspended them when beginning to be apparent, and consequently appear to have prevented the absorption of the poison. The experiments of Dr. Barry have not only been confirmed by repetition, but others have been performed with the white oxyde of arsenic, hydrocyanic acid, and the upas tieuté.

1. Eight grains of white arsenic were introduced into a wound made in the thigh of a dog, three quarters of an hour after a cupping glass was applied to the wound, and kept on for four hours, and the animal suffered no inconvenience. Another dog under the same circumstances, where the cupping glass was not applied, died at the end of fifteen hours.

2. Six drops of hydrocyanic acid were poured into a little wound made in the thigh of a rabbit, the cupping glass was applied for twelve minutes, and the animal shewed no signs of having been poisoned; but when it was taken away convulsions came on so suddenly that it was thought to be dead, but a fresh application of the cupping glass restored it to its former state of tranquillity; the same effects ensued upon removing it again, and it was only after half an hour after the introduction of the poison that it could be removed with impunity. Another rabbit, treated with the same quantity of acid, where no cupping glass was used, died in two minutes. The results of trials made with a grain of the Upas Tieuté were in all respects similar. Dr. Barry concludes that the cessation of the symptoms of poisoning from the application of the cupping glasses, arising in consequence of that portion of the poison which has been absorbed being recalled to the wound and taken out of the circulation,—a position which is combated by M. Segalas, who believes that the cupping glass only acts by preventing the absorption of any new quantity of the poison, and that the portion which has penetrated ceases to act because it is rejected by the different excretions. (*Archives Generales,* Octobre.)

5. *Cauterization of the Pustules of the Small Pox.*—We formerly mentioned that a very warm discussion upon this subject has been going on in Paris, from which it appears that many practitioners altogether deny the utility of this plan, urging that the pustules continue their progress commonly under the eschar, and in spite of it; whilst others go beyond this, and say that cerebral inflammation is too often the consequence of this method. The claim of priority in the use of caustic is contested between MM. SERRES and BRETONNEAU; but it appears that there is a decided difference in the manner of employing the remedy between those gentlemen. M. Serres uses a solution of lunar caustic in water, of from twenty to thirty-five grains to the ounce, with which the pustules are washed; whilst M. Bretonneau applies the solid caustic to each pustule separately, after opening them with a lancet. It appears further, from the observations of M. GAZO, that this plan is only successful when adopted immediately upon the appearance of the pustules; after the fourth day, they will continue their progress uninfluenced by the application. (*Revue Medicale*, Octobre.)

6. *Effects of Iodine.*—M. LOCHER-BALBER has published several cases, in which the good effects of the above medicine were demonstrated. The first case is that of a woman, twenty-five years of age, who, otherwise of a good constitution, was subject at each period of menstruation to violent head-aches, so as to be obliged to keep her bed; sometimes violent pains in the teeth, or bowels, occurred instead of head-ache. After taking half an ounce of the tincture of iodine, (the dose is not mentioned,) she was freed of all her ailments, and the menses have subsequently been quite regular, and not preceded by any painful affection. The second and third cases are so far similar, inasmuch as they relate to symptoms dependent upon menstruation, and they equally yielded to the tincture of iodine. Three other cases are recorded of enlarged lymphatic glands, in one the external and internal use of iodine produced no effect on the disease, in the other two cases a cure was effected. M. Locher-Balber finally relates the following case:—A child, six years of age, affected with tinea for a long time, had enlarged glands of the neck, the least of which were as large as a nut. Five drops of tincture of iodine were given three times a-day, the swelling of the glands diminished considerably, and the tinea was radically cured. The medicine was obliged to be withheld sometimes, because it occasioned, after a certain period, a feeling of great heat in the stomach: the patient was removed from the author's care, and therefore he does not know the termination of the case. Other cases relate to the ill effects of the tincture of iodine; and in one instance a general emaciation ensued, and the remedy was abandoned. (*Ibid.*)

7. *Muriate of Gold in Syphilis.*—Dr. GUSTAVUS BENABEN has published some cases of the above disease cured by the muriate of gold. He precedes the relation of these cases by reminding us that these remedies are as ancient as the year 1540, and that they have been recommended at various periods down to the time of Dr. CHRETIEN, whose preparation, viz. the muriate of gold and soda, is employed by the author. We only quote him in order to inform such of our readers as are disposed to put this remedy to the test of experience, that it is usually employed in frictions on the tongue, beginning with the fourteenth part of a grain, and gradually increasing it to the eighth; sometimes it is also administered in very minute doses internally: copious sweats, or a great flow of urine, are the effects of the medicine on the animal economy. The quantity required for the use of secondary symptoms appears to have been from six to twelve grains. *(Ibid.)*

---

### SURGERY.

8. *Calculus in the Urethra.*—M. Boulu relates the following case:—A boy, five years of age, had, for two years, experienced slight attacks of hæmaturia, which were frequently renewed: there was, however, no other symptom denoting the presence of calculus; there was no pain in the bladder, nor whilst passing the water, nor even when there was a discharge of blood. His parents, however, were uneasy at this circumstance, and medical means were resorted to, without effect. In the night of the 7th of May, he was suddenly attacked by retention of urine; and, on the following morning, Dr. Boulu was called to see the patient. He was in a state of extreme suffering, making forcible, but useless, efforts to pass urine; the belly swollen; the face flushed, with a good deal of heat and fever. The introduction of the catheter was, of course, the first thing that appeared necessary to be done; but the child screamed so violently when the attempt was made, that his parents begged the Doctor to desist. Yielding with regret to their solicitations, the warm bath, with fomentations and enemata, were prescribed.

At eight o'clock in the evening, the Doctor was again sent for. None of the above means, though rigidly followed, had been attended with any good effect. The boy was senseless, and affected by convulsions, with but little interval between each; the belly was enormously distended; and, upon making an attempt to pass the catheter, it was stopped by some foreign body: the extraction of this substance, which was considered to be a calculus, was therefore inevitable; and, as M. Boulu had neither the forceps of Hunter nor Maigny at hand, instead of losing time in endeavouring to procure these instruments, he made an incision into the urethra, drawing the skin towards the glans, to prevent this incision being parallel with that in the urethra. This first incision was five lines in length, and only included the skin. The edges being separated, the urethra was opened a little obliquely, and a calculus extracted: a little blood escaped. The child recovered from its state of insensibility during the operation; and, as soon as the stone was removed, a quantity of urine escaped, first of a red colour: latterly a little escaped by the wound, which was dressed simply, without the introduction of any catheter.

The next day, every thing went on well: much urine had passed through the penis, and very little by the wound. On the third day, all danger had disappeared: not a drop of water escaped by the wound, which is now entirely healed. *(Ibid.)*

### MISCELLANEOUS.

9. *Society for the Diffusion of Obstetrical Knowledge.*—It is proposed to establish a "Society for the Diffusion of Obstetrical Knowledge."

The Society to consist of all denominations of medical practitioners resident in Great Britain, whether physicians, surgeons, or apothecaries, legally authorised to practise as such, who exercise or have exercised the art of midwifery. The number to be limited to 500, and to be divided into two classes, of resident and non-resident members.

The management of the Society to be vested in one president, two vice-presidents, one treasurer, a directing committee of ten, and a conservator of the museum, who shall act also as librarian.

All members to pay three guineas on their admission; and the annual subscription to be two guineas for the resident, and one guinea for the non-resident members.

The Society to have a house in a central part of the town; with a museum and library, open all the year round for a certain number of hours daily, at which members may attend to consult books, write, or converse together in the manner of the best clubs.

The instructions and orders of the officers and committee to be executed by an honorary and a stipendiary secretary; the latter of whom shall also be sub-conservator and sub-librarian, and shall reside in the house.

The members to present a book or books, whether modern or ancient, on subjects of medicine, particularly midwifery, or their own works, for the purpose of forming a library; and also all such preparations of healthy or morbid anatomy, illustrative of the different branches of obstetrical science, as they may be able to spare.

An ordinary general meeting of the members to take place every Monday evening, at eight o'clock during the months of November, December, January, February, March, and April, for the purpose of discussing obstetrical subjects, and conducting the ordinary affairs of the Society. An anniversary general meeting to be held on the first Monday in April, for the election of officers, and for receiving the committee's and treasurer's Reports.

Two prizes, value twenty-five guineas each, to be funded, for the best papers on subjects of obstetrical science, and its various branches: the prizes to be distributed by the president at the anniversary general meeting.

A Bulletin of the proceedings of the Society, drawn up in an instructive form, and in the manner of the Scientific Gazette, with occasional wood-cuts and lithographic engravings, to be published on the first of every month, while the ordinary meetings are held, and distributed gratuitously to the contributing resident and non-resident members.

The principal objects of the Society to be—1. To promote the diffusion of obstetrical knowledge. 2. To encourage public and private schools of midwifery. 3. To procure some legislative enactment, to regulate the practice of midwifery in Great Britain. 4. To form a plan for the gradual education of a certain number of respectable females, to practise as midwives in those parts of the country where male practitioners cannot be procured. 5. To suggest to the public the best method of securing protection and a proper treatment to the children of those females who go out as wet nurses, by forming an establishment for the temporary reception of such children.

*** We understand that a meeting has taken place, and that some of the leading practitioners in midwifery have signified their intention of becoming members of this Society.—EDITORS.

10. *Note from* DR. ELLIOTSON *to* DR. MACLEOD, *respecting the Carbonate of Iron.*

Dear Sir,—Accept my best thanks for pointing out, in the last number of your Journal, an error which I have committed in my paper upon the Subcarbonate of Iron, with respect to the largest quantity of that medicine exhibited by Mr. Hutchinson. I stated that the largest quantity found necessary by him is four scruples and a half in the twenty-four hours, and concluded that this was the largest quantity he ever employed; whereas, by consulting his work, I find that he gave three or four scruples twice a-day. Even by consulting my own note-book, in which I make abstracts of the books I read, I find written, "The largest quantity given by Mr. Hutchinson is four scruples twice a-day."—How the mistake originated, I cannot imagine, unless from a bad habit of preparing for the press upon loose slips of paper, and a reference to some other medicine and some other author having slipped among my notes upon the Subcarbonate of Iron, and being mistaken by me as referring to Mr. Hutchinson.

The mistake, I must confess, appears to me perfectly immaterial; because if Mr. Hutchinson had been able to cure Tic douloureux with three scruples and a half a-day, it would have been a better thing than to cure it with three or four scruples twice a-day; and because the quantity of half an ounce, which I have shewn may be commonly given four or six times in the twenty-four hours, is equally unexpected whether three scruples and a half had been given by others in the twenty-four hours, or four scruples twice in that time.

That my error was unintentional, must appear not only from these circumstances, but from my having said in the same sentence, that some practitioners give a drachm three times a-day; ("the ordinary dose is from fifteen to thirty grains two or three times in the twenty-four hours, but some practitioners occasionally give a drachm,") which is a scruple more than Mr. Hutchinson ever found it necessary to exhibit. Still I have committed an error, and regret it. There is no necessity for me to remark how much the profession and society are indebted to Mr. Hutchinson for his discovery of the utility of the Subcarbonate of Iron in Tic douloureux; and I have expressly declared in my paper, that I should not have thought of exhibiting it in Chorea and Paralysis agitans, but for its utility in Tic douloureux.

I beg you will make whatever use you think proper of this note; and, indeed, you could not oblige me more than by giving it a place in your next number.—I remain, dear Sir, your's faithfully,

*Grafton-street, Dec. 3, 1825.* J. ELLIOTSON.

P.S.—The edition consulted by me of Mr. Hutchinson's work is the first: I have been unable to procure the second.

### METEOROLOGICAL JOURNAL,

By Messrs. William Harris and Co. 50, Holborn, London.

*From November 20 to December 19, 1825.*

| | Moon. | Rain gauge | Therm. | | | Barom. | | De Luc's Hyg. | | Wind. | | Atmospheric Variations. | | |
|---|---|---|---|---|---|---|---|---|---|---|---|---|---|---|
| | | | 9 A.M. | Max. | Min. | 9 A.M. | 10 P.M. | 9 A.M. | 10 P.M. | 9 A.M. | 10 P.M. | 9 A.M. | 2 P.M. | 10 P.M. |
| Nov | | | | | | | | | | | | | | |
| 20 | | | 38 | 43 | 43 | 30.06 | 30.01 | 80 | 80 | WNW | SW | Fine | Fine | Fine |
| 21 | | | 53 | 53 | 38 | 29.68 | 29.72 | 85 | 76 | WSW | W | | Rain | |
| 22 | | .22 | 40 | 45 | 34 | 29.76 | 30.02 | 76 | 75 | WNW | NW | | Fine | |
| 23 | | | 40 | 43 | 47 | 30.16 | 30.05 | 75 | 90 | WSW | WSW | | | Sleet |
| 24 | | .04 | 49 | 51 | 41 | 30.08 | 29.87 | 93 | 95 | W | W | Foggy | Rain | Cloud. |
| 25 | ○ | | 43 | 46 | 38 | 30.14 | 30.22 | 84 | 77 | WNW | W | Fine | Fine | Fine |
| 26 | | | 45 | 53 | 40 | 29.97 | 29.63 | 78 | 75 | SW | SW va. | | Rain | Cloud. |
| 27 | | | 46 | 51 | 50 | 29.85 | 29.62 | 67 | 85 | W | SSW | | Fine | Rain |
| 28 | | .29 | 44 | 53 | 42 | 29.21 | 29.08 | 87 | 84 | SW | SW | Rain | | Fine |
| 29 | | | 43 | 53 | 40 | 28.90 | 28.81 | 78 | 80 | SW | SW | Fine | | |
| 30 | | | 39 | 40 | 32 | 29.30 | 29.58 | 80 | 78 | N | N | | | |
| Dec | | | | | | | | | | | | | | |
| 1 | | | 32 | 35 | 38 | 29.51 | 29.83 | 85 | 77 | E | SE var. | | | |
| 2 | | | 44 | 45 | 40 | 28 84 | 28.96 | 91 | 82 | W var. | SW | Rain | Cloud. | |
| 3 | ☾ | .71 | 40 | 45 | 39 | 29.10 | 29.12 | 85 | 85 | SW | SW | Fine | Fine | Foggy |
| 4 | | | 39 | 41 | 33 | 29.11 | 29.31 | 86 | 85 | N | N | Rain | Rain | Fine |
| 5 | | | 35 | 41 | 44 | 29.26 | 29.18 | 94 | 95 | NE | SSW | | | Rain |
| 6 | | .96 | 46 | 48 | 40 | 29.29 | 29.24 | 91 | 95 | SW | ESE | Fine | Fine | Fine |
| 7 | | | 43 | 51 | 43 | 29.11 | 29.21 | 92 | 85 | E | E | | | |
| 8 | | | 44 | 45 | 44 | 29.27 | 29.23 | 92 | 95 | ENE | NE | | | |
| 9 | ● | | 43 | 45 | 43 | 29.30 | 29.45 | 92 | 85 | NE | NNE | | | |
| 10 | | | 43 | 45 | 42 | 29.52 | 29.61 | 88 | 85 | NW | W | | | |
| 11 | | | 43 | 46 | 39 | 29.64 | 29.72 | 87 | 87 | W | WNW | | | |
| 12 | | | 39 | 42 | 35 | 29.75 | 29.71 | 91 | 95 | SW | NW | Foggy | Foggy | Foggy |
| 13 | | | 35 | 45 | 45 | 29.67 | 29.57 | 95 | 95 | WSW | SSW | | Fine | Rain |
| 14 | | | 47 | 50 | 37 | 29.18 | 29.16 | 95 | 85 | SW va. | SW | Rain | Rain | Cloud. |
| 15 | | .23 | 37 | 42 | 45 | 29.43 | 29.66 | 80 | 79 | W | SW | Fine | Fine | |
| 16 | | | 47 | 50 | 48 | 29.68 | 29 72 | 75 | 79 | WSW | SW | | | Fine |
| 17 | ☽ | .27 | 51 | 52 | 45 | 29.56 | 29.71 | 95 | 90 | SW | WSW | Rain | | |
| 18 | | | 50 | 52 | 48 | 29.54 | 29.45 | 88 | 89 | SSW | SSW | Fine | | Rain |
| 19 | | .12 | 49 | 49 | 41 | 29.24 | 29.33 | 88 | 79 | S | SSW | Rain | Cloud. | Fine |

The quantity of rain fallen in the month of November, was 2 inches and 18.100ths.

